# Molecular Markers of Sulfadoxine-Pyrimethamine Resistance in Samples from Children with Uncomplicated Plasmodium falciparum at Three Sites in Angola in 2019

**DOI:** 10.1128/aac.01601-22

**Published:** 2023-03-14

**Authors:** Stefano R. Rosillo, Pedro Rafael Dimbu, Ana Luisa M. Cândido, Je-Hoon Michael Oh, Carolina Miguel Ferreira, Benjamin Nieto Andrade, Sarah Labuda, Roberta Horth, Julia Kelley, Joana F. M. Morais, Filomeno Fortes, José Franco Martins, Eldin Talundzic, Mateusz M. Pluciński

**Affiliations:** a Malaria Branch, U.S. Centers for Disease Control and Prevention, Atlanta, Georgia, USA; b National Malaria Control Program, Ministry of Health, Luanda, Angola; c National Institute of Health Research, Ministry of Health, Luanda, Angola; d Population Services International Angola, Luanda, Angola; e United States President’s Malaria Initiative, U.S. Centers for Disease Control and Prevention, Luanda, Angola; f Institute of Hygiene and Tropical Medicine, Nova University of Lisbon, Lisbon, Portugal; g United States President’s Malaria Initiative, Malaria Branch, U.S. Centers for Disease Control and Prevention, Atlanta, Georgia, USA

**Keywords:** *dhfr*, *dhps*, surveillance

## Abstract

Sulfadoxine-pyrimethamine (SP) is used for prevention of malaria in pregnant women in Angola. We sequenced the Plasmodium falciparum dihydrofolate reductase (*pfdhfr*) and dihydropteroate synthase (*pfdhps*) genes, implicated in SP resistance, in samples collected during a 2019 study of artemisinin-based combination therapy efficacy in Benguela, Lunda Sul, and Zaire provinces. A total of 90 day 0 and day of failure samples were individually sequenced, while 508 day 0 samples from participants without recurrent parasitemia were pooled after DNA extraction into 61 pools. The N51I, C59R, and S108N *pfdhfr* mutations and A437G *pfdhps* mutations were present at high proportions in all provinces (weighted allele frequencies, 62% to 100%). The K540E *pfdhps* mutation was present at lower proportions (10% to 14%). The A581G *pfdhps* mutation was only observed in Zaire, at a 4.6% estimated prevalence. The I431V and A613S mutations were also only observed in Zaire, at a prevalence of 2.8% to 2.9%. The most common (27% to 66%) reconstructed haplotype in all three provinces was the canonical quadruple *pfdhfr pfdhps* mutant. The canonical quintuple mutant was absent in Lunda Sul and Benguela and present in 7.9% of samples in Zaire. A single canonical sextuple (2.6%) mutant was observed in Zaire Province. Proportions of the *pfdhps* K540E and A581G mutations were well below the World Health Organization thresholds for meaningful SP resistance (prevalence of 95% for K540E and 10% for A581G). Samples from therapeutic efficacy studies represent a convenient source of samples for monitoring SP resistance markers.

## INTRODUCTION

Artemisinin-based combination therapy (ACT) became the first-line treatment for uncomplicated malaria in 2006 in Angola, reaching full national implementation between 2007 and 2008 (1). Prior to that, the treatments used included chloroquine, amodiaquine, and sulfadoxine-pyrimethamine (SP) (2). Even after the change to ACTs for therapeutic use, SP continues to be administered to pregnant women for intermittent preventive treatment (IPTp-SP) during antenatal care services (ANC). Angola follows World Health Organization (WHO) guidelines, which state that IPTp-SP can be given as early as the second trimester, with doses given at least 1 month apart during the four recommended ANC visits (3). It is recommended that women receive three or more doses of SP as IPTp, as this is associated with higher mean birth weight, fewer low birth weight infants, and a reduction in placental malaria compared to those receiving two IPTp doses or fewer (4). Despite this recommendation, the 2015–2016 Demographic and Health Survey for Angola showed that only 19% of pregnant women were receiving three or more IPTp doses (5).

Plasmodium falciparum resistance to SP occurs as the result of a combination of single-nucleotide polymorphisms (SNPs) at specific codons in the genes encoding dihydrofolate reductase (*pfdhfr*) and dihydropteroate synthase (*pfdhps*), two enzymes involved in the folate biosynthesis pathway. Resistance to SP can be inferred from the cumulative number of mutations at codons 16, 51, 59, 108, and 164 in *pfdhfr* and codons 431, 436, 437, 540, 581, and 613 in *pfdhps* (6–11). Parasite genotypes are therefore typically defined by the number of mutations present in the genes. Several genotypes have been identified as notable and have been characterized and tracked in the SP resistance surveillance literature. The canonical quintuple mutant (IRN ISGEAA) with *pfdhfr* mutations N51I, C59R, and S108N and *pfdhps* mutations A437G and K540E has been associated with clinical and parasitological SP treatment failure and has been shown to reduce the prophylactic period (6, 7). An additional mutation within *pfdhps* at A581G defines the sextuple mutant (IRN ISGEGA), which has been associated with decreased efficacy of IPTp-SP, resulting in low infant birth weight and increased placental inflammation (12–14). Additionally, a recent large study in seven West African countries implementing seasonal malaria chemoprophylaxis with SP plus amodiaquine showed strong evidence of selection of the VAGKGS *pfdhps* haplotype, characterized by the rare I431V mutation, during the study period (15), although its exact effect on parasite susceptibility to SP is still unknown.

In 2013, a WHO Evidence Review Group recommended that countries consider discontinuing IPTp-SP once the population prevalence of the *pfdhps* 540E mutation is >95% and the prevalence of the *pfdhps* 581G mutation is >10%, as SP is unlikely to be effective in the presence of these mutations (4). However, current WHO guidelines do not recommend withdrawing IPTp-SP even when there is a high prevalence of mutations associated with SP resistance (16).

Although current data on SP resistance in Angola are limited, surveillance of SP resistance in Angola dates to the early 1990s (1), with the earliest molecular studies dating to the 2000s. Initial molecular studies were restricted to an investigation of a limited number of SNPs and limited scope. A seminal five-province survey conducted in 2007 in Huambo, Cabinda, Uíge, Kwanza Norte, and Malanje provinces mapped the distribution of the N51I, C59R, and S108N *pfdhfr* mutations and the A437G and K540E *pfdhps* genes using restriction fragment length polymorphism and showed high proportions (>83%) of the N51I, S108N, and A437G mutations (17). However, the proportions of C59R (49%) and K540E (13%) were lower. A Sanger sequencing study in the interior of Benguela Province in 2010 to 2011 also found low proportions of C59R (26%) and no K540E mutations (18). A more recent study in 50 patients in one hospital in the exclave province of Cabinda in 2018 confirmed continued high proportions of N51I, S108N, and A437G using Sanger sequencing but also found 94% prevalence of C59R and 31% prevalence of K540E in this hospital cohort; when considering haplotypes, 37.5% were quintuple mutants, and a single sample had six mutations, although not the six that define the canonical sextuple mutant (19).

Molecular surveillance for antimalarial resistance is a component of the national Angolan malaria control strategy. The country implements therapeutic efficacy studies (TESs) every 2 years at three fixed sentinel sites to assess ACT effectiveness. Samples from these studies present an opportunity to screen for known markers of antimalarial resistance, including those associated with resistance to ACTs (20), but also markers for other drugs, such as SP. The use of next-generation sequencing methods (NGS) allows high-throughput screening of a large number of samples, including the use of pooling to reduce time and sequencing costs (21).

Here, we used individual and pooled targeted amplicon deep sequencing screening methods to genotype samples from the 2019 Angola TES to determine their SP resistance genetic profiles.

## RESULTS

**FIG 1:**
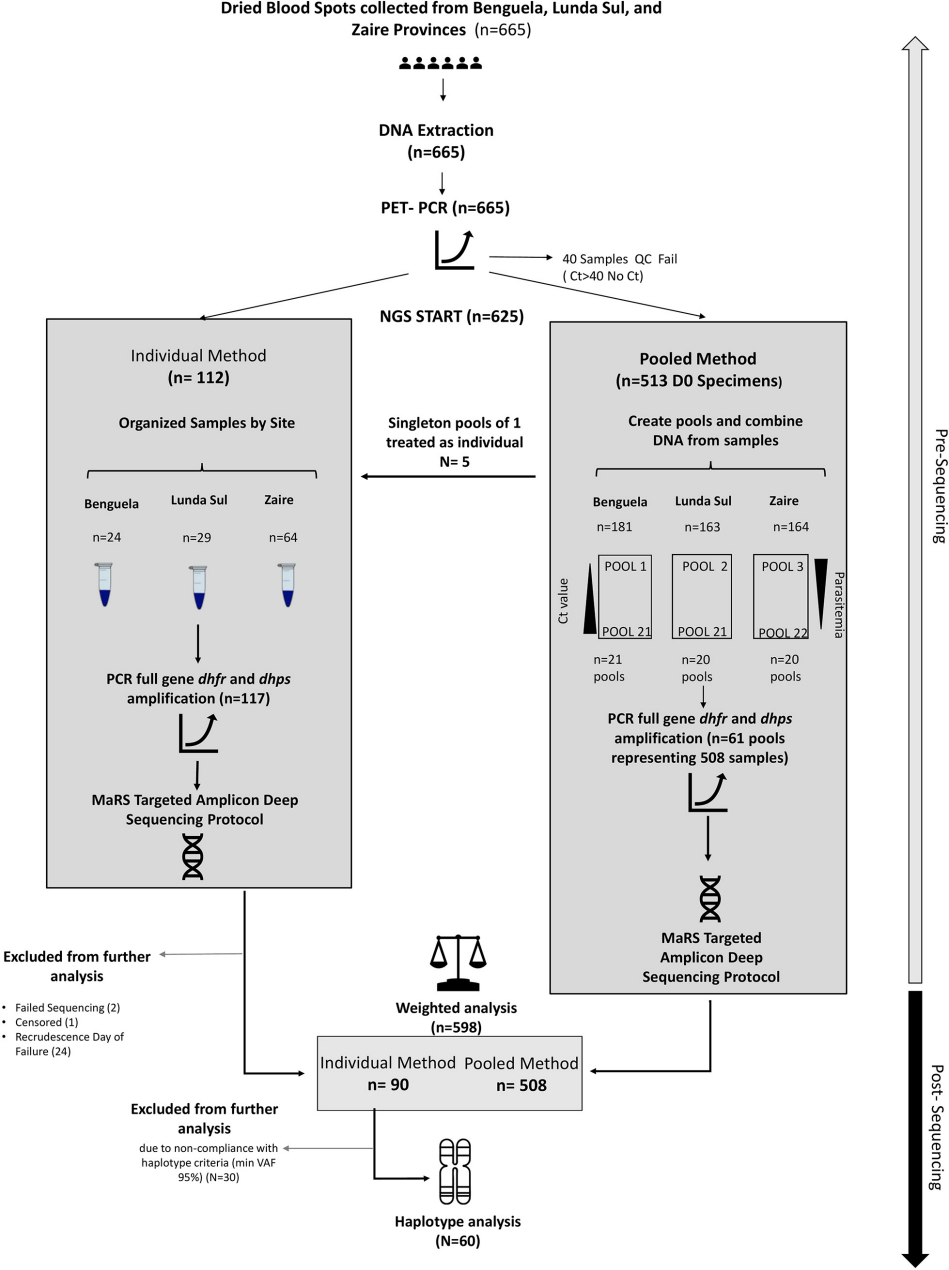
Laboratory workflow for combined individual and pooled targeted amplicon sequencing analysis of dried blood spots from therapeutic efficacy monitoring in Benguela, Lunda Sul, and Zaire provinces in Angola, 2019. PET-PCR, photo-induced electron transfer PCR; MaRS, Malaria Resistance Surveillance; VAF, variant allele frequency.

Out of the initial 665 samples, 40 (6%) samples failed the initial quality control check due to a cycle threshold (*C_T_*) value of >40 or no *C_T_* (Fig. 1). Of the remaining 625 samples, 112 paired day 0 (D0) and day of failure (DoF) samples were identified for individual analysis, and 513 were directed for the pooled analysis. A total of 61 pools were constructed (Fig. 1; see also Fig. S1 in the supplemental material), representing 508 samples; the remaining 5 samples had outlier *C_T_* values that prevented them from being pooled with other samples, and these samples were analyzed individually. As a result, 117 samples were analyzed in the individual sample pipeline. Of these 117 samples, 2 failed sequencing, 1 was excluded as the patient was censored from the analysis due to a non-*falciparum* infection, and 24 were excluded from further analysis since they were DoF samples from recrudescences, resulting in 90 total samples for the individual analysis (Fig. 1; Table 1). All 61 pools were successfully sequenced, resulting in a total of 508 samples providing data for the pooled analysis.

**TABLE 1:** Sample size for individual and pooled analysis of molecular markers of resistance at 3 sites in Angola in 2019

Parameter	Data from site in:			Total
	Benguela	Lunda Sul	Zaire	
No. of individual samples	21	17	52	90
Day 0	12	15	33	60
ACPR or censored	2	1	3	6
Treatment failure	10	14	30	54
Day of failure	9	2	19	30
Early treatment failure	0	0	1	1
Reinfection	9	2	18	29
No. of pooled day 0 samples	181	163	164	508
No. of pools	21	20	20	61
Median pool size (range)	10 (3–10)	10 (3–10)	10 (2–10)	10 (2–10)

In the combined weighted analysis, all the N51I and S108N mutations in *pfdhfr* were near fixation at all three sites, while the frequency of the C59R mutation in the samples from children at the three sentinel site clinics ranged from 62% to 90% (Table 2). For *pfdhps*, the A437G mutation was present at high levels near fixation in all three provinces, ranging from 90% in Lunda Sul to 100% in Benguela (Table 3). However, the key K540E mutation, present in the canonical quintuple mutant, was present at much lower levels, ranging from 10% to 14%. The defining mutation in the canonical sextuple mutant, A581G, was present at even lower levels, entirely absent in Benguela and Lunda Sul, and at a level of 4.6% in Zaire. The I431V mutation was only found in Zaire, at an estimated frequency of 2.9%. Similarly, the A613S mutation was also only found in Zaire at a frequency of 2.8%.

**TABLE 2:** Frequency of mutations in *pfdhfr* at 3 sites in Angola in 2019

Mutation	Data from site in:					
	Benguela		Lunda Sul		Zaire	
	*n*	%VAF^*a*^	*n*	%VAF	*n*	%VAF
A16V	195	0	180	0	216	0
N51I	201	93	180	89	216	100
C59R	201	66	179	62	216	90
S108N	202	100	180	100	216	100
I164L	201	0	179	0	216	0

a VAF, variant allele frequency.

**TABLE 3:** Frequency of mutations in *pfdhps* at 3 sites in Angola in 2019

Mutation	Data from site in:					
	Benguela		Lunda Sul		Zaire	
	*n*	%VAF^*a*^	*n*	%VAF	*n*	%VAF
I431V	201	0	180	0	215	2.9
S436A	201	9.4	180	3.9	214	17
A437G	201	100	180	90	214	99
K540E	198	14	179	13	214	10
A581G	200	0	179	0	215	4.6
A613S	201	0	179	0	216	2.8

a VAF, variant allele frequency.

When constructing *pfdhfr pfdhps* haplotypes from the 90 individual samples, a total of 60 (67%) profiles were able to be reconstructed according to the prespecified criteria for single-strain infections. The most common *pfdhfr pfdhps* haplotype was the canonical quadruple IRN ISGKAA mutant (Table 4), present across the three sites, with a frequency varying from 27% to 66%. Three canonical quintuple mutants (IRN ISGEAA) were identified, all in Zaire, at a frequency of 7.9%. One canonical sextuple mutant (IRN ISGEGA) and one instance of the emerging IRN VAGKGS mutant were found in Zaire, both at an estimated frequency of 2.6%.

**TABLE 4:** Frequency of *pfdhfr* and *pfdhps* haplotypes at 3 sites in Angola in 2019

Haplotype^*a*^	No. of mutations	No. (%) of occurrences at site in:		
		Benguela	Lunda Sul	Zaire
ICN ISAKAA	2	0	1 (9.1)	0
IRN ISAKAA	3	0	1 (9.1)	0
ICN ISGKAA	3	2 (18)	2 (18)	1 (2.6)
NRN ISGKAA	3	0	2 (18)	0
IRN IAAKAA	4	0	0	1 (2.6)
IRN ISGKAA	4	7 (64)	3 (27)	25 (66)
ICN IAGKAA	4	0	2 (18)	0
ICN ISGEAA	4	0	0	1 (2.6)
IRN IAGKAA	5	2 (18)	0	5 (13)
IRN ISGEAA	5	0	0	3 (7.9)
IRN ISGEGA	6	0	0	1 (2.6)
IRN VAGKGS	8	0	0	1 (2.6)
Total		11 (100)	11 (100)	38 (100)

a Haplotypes were defined at *pfdhfr* codons *51*, *59*, and *108* and *pfdhps* codons 431, 436, *437*, *540*, *581*, and 613 (italic numbers denote the mutations used to define the canonical quintuple and sextuple mutant haplotypes). Wild type: NCS ISAKAA.

## DISCUSSION

We used a mixed individual and pooled targeted amplicon deep sequencing approach to evaluate the frequency of genotypic markers of SP resistance using samples collected from children at the three sentinel site clinics during a therapeutic efficacy study. We found that across all three provinces, the N51I and S108N *pfdhfr* mutations were near fixation. The C59R *pfdhfr* mutation was also present at high levels. The K540E *pfdhps* mutation was present at low levels (≤14%). The A581G *pfdhps* mutation was absent in Benguela and Lunda Sul and present in 4.6% of samples at the Zaire site. Among the samples for which haplotypes were able to be inferred, the canonical quadruple *pfdhfr pfdhps* mutant was the most prevalent in all sites. The canonical quintuple mutant was absent in Lunda Sul and Benguela and present in 7.9% of samples in Zaire. One canonical sextuple mutant was reconstructed in Zaire Province.

Notably, the I431V *pfdhps* mutation was detected at a 2.9% frequency in Zaire Province, although it was absent in the other two provinces. This pattern of frequency almost exactly matched that of A613S, which was present at a frequency of 2.8% in Zaire Province and was absent in the other provinces. This confirms reports from West Africa that suggest that the I431V mutation often occurs together with A613S but not K540E (11, 15). Consistent with this finding, the only reconstructed *pfdhps* haplotype containing the I431V mutation was VAGKGS, which carries the A613S polymorphism but not K540E. This haplotype is the same one that was observed to be increasing in frequency in countries using SP plus amodiaquine for large-scale chemoprophylaxis in West Africa (15). The appearance of this haplotype in central Africa, far from the nexus of seasonal chemoprevention in the Sahel, warrants further vigilance.

These are the first reported data for SP resistance markers in Lunda Sul and Zaire provinces, precluding any analysis of trends at these sites. A single previous study in Benguela Province had been conducted in 2010 to 2011 in the interior of the province, roughly 150 km away from the TES site in the capital city of Benguela (18). Our current study found higher proportions of the C59R mutation (66% versus 26%) and also identified the presence of K540E mutations, which were absent in the 2010–2011 study. Changes in the frequency of SP resistance markers are likely affected by selection pressure by continued exposure of parasite populations to SP. There is a paucity of recent data on SP usage in Angola. The most recent household survey in 2015 to 2016 showed that only 19% of pregnant women had received three or more IPTp doses (5), a moderate increase over a very low baseline of 1.4% in 2006 to 2007 (22). The same 2015–2016 survey also showed that 8% of children treated with an antimalarial had received SP, evidence of some residual use of SP for treatment. However, a 2016 health facility survey did not uncover any prescription of SP for treatment in the public sector (23).

A recent molecular resistance mapping study of SP markers in the Democratic Republic of Congo found a nationwide east-west gradient in the frequency of the K540E mutation, with lowest proportions in the eastern half of the country (24). These findings are consistent with our observed low proportions of the K540E mutation in the three provinces studied here, two of which (Lunda Sul and Zaire) border the Democratic Republic of Congo.

The pooling strategy we employed saves time by reducing the number of PCRs and separate library preparation reactions necessary. Specifically, in the pooled arm, we were able to reduce the number of gene amplification steps and library preparation steps from 508 to 61. As such, this strategy allows for a high-throughput method of estimating allele frequencies from large data sets. It is particularly applicable when individual sample data, for example parasite phenotypes, are not important for interpretation of data. For example, we were able to pool day 0 samples from the original TES because there was no additional relevant individual sample information, as the TES did not include any SP treatment. Samples from participants with recurrent parasitemia were individually sequenced only because other markers, including ones associated with the study drugs, were also included in the targeted amplicon sequencing panel, necessitating the ability to assign genotypes to individual samples with a given phenotype. Nevertheless, this strategy of mixed individual and pooled data can still yield appropriate estimates with the weighting strategy we employed here.

There are, however, limitations to the pooling approach. First, samples can contribute different amounts of DNA to a pool, and distinct parasitic genotypes occur concurrently in each individual sample (multiclonal infections), leading to biased estimates of allele frequencies at the pool level, especially if there is differential amplification by sample or strain. We attempted to minimize the DNA quantity bias by only pooling samples with similar *C_T_* value. Second, construction of haplotypes is generally not possible with pooled data, since mutations cannot be linked across samples. Due to this, we were only able to reconstruct haplotypes for the individually sequenced samples. Notably, we had strict criteria for reconstructing haplotypes, only reporting on samples with major allele frequencies greater than 95% to avoid spurious haplotypes arising from mixed infections. This level of quality control is rare in the SP molecular surveillance literature, and comparison of our results with other haplotype prevalence estimates should account for this.

Samples collected during therapeutic efficacy trials represent a convenient source for monitoring molecular markers of resistance. Therapeutic efficacy sentinel sites are often fixed and thus allow tracking of trends in the frequency of molecular markers of resistance if multiple rounds are analyzed in turn. Moreover, the samples are from well-characterized, confirmed P. falciparum infections with sufficiently high parasite densities to ensure likely sequencing success. Typically, samples are sequenced for molecular markers of resistance associated with the antimalarial included in the trial. However, NGS approaches allow the inclusion of additional markers at low marginal cost. As such, including SP resistance markers as part of this screening is easy to justify, especially given the relatively scant data on these markers in many countries of endemicity. However, caution should be taken in generalizing the findings from sequencing samples collected during therapeutic efficacy trials. For example, in this study, children from the catchment areas of two health clinics in each of three provincial capital cities were enrolled in the trials. It is unknown if the frequency of SP resistance markers in the samples from these children is representative of the true prevalence in the overall circulating parasite population. Finally, the clinical resistance of all the *pfdhfr* and *pfdhps* mutants observed in this study has not been fully elucidated, so their true resistance phenotype is still unknown.

Our findings suggest that SP is still suitable for chemoprophylactic use at these three sites in Angola, since the K540E mutation was found at low proportions (10% to 14%), and the A581G mutation was absent from two sites and present at only a 4.6% estimated frequency in Zaire. However, future studies should continue to monitor for changes in SP resistance profiles. The use of TES samples will allow for assessment of trends at the three fixed sentinel sites.

## MATERIALS AND METHODS

### Study design.

A retrospective analysis was conducted on molecular markers associated with SP resistance from samples collected from a 2019 prospective clinical outcome trial in Angola (25). Two drugs, artemether-lumefantrine and artesunate-amodiaquine, were tested at each of the three sites: M’Banza Congo, Zaire Province; Saurimo, Lunda Sul Province; and Benguela, Benguela Province.

### Sample collection and pooling strategy.

Samples came from children aged 6 months to 12 years at the three sentinel site clinics with uncomplicated P. falciparum monoinfection presenting as febrile illness that met the standard WHO *in vivo* TES inclusion criteria (25). The children were treated with artemether-lumefantrine or artesunate-amodiaquine and followed for 28 days to assess the clinical and parasitological response. Blood spots were collected on a Whatman 903 filter on day 0 (D0) and during follow-up visits after day 2, dried, and stored in individual Ziploc bags with a desiccant. Baseline D0 samples from all participants and day of failure (DoF) samples from participants with recurrent parasitemia were included in the molecular analysis.

### DNA extraction.

Six 3-mm punches from dried blood spots (DBS) were used to extract genomic DNA using a DNA extraction minikit following the manufacturer’s instructions (Qiagen, Hilden, Germany). The DNA was eluted in 150 μL of elution buffer and stored at −20°C until use.

### Parasitemia estimation using PET-PCR and pooling.

Following DNA extraction, photo-induced electron transfer PCR (PET-PCR) was performed on all 665 samples in duplicate (26). Any samples with a cycle threshold (*C_T_*) value of >40 or no *C_T_* value present were excluded from next-generation sequencing (NGS). A total of 61 pools were constructed from 508 D0 nonfailure samples (Fig. 1). For each province, the D0 nonfailure samples were aligned from lowest to highest *C_T_* and binned into pools of up to 10 samples maximum each, such that all samples in a pool differed by less than 1.0 *C_T_* value (see Fig. S1 in the supplemental material) (21). Samples not within 1 *C_T_* value of any other samples were individually sequenced and later represented in the individual analysis. DNA (2 μL) from each sample was combined to create a pooled DNA lot for each pool.

### PCR enrichment of *pfdhps* and *pfdhfr*.

PCRs were performed to amplify the full-length *pfdhfr* and *pfdhps* genes for the individual patient samples and the pools using a previously described protocol (27). The P. falciparum HB3, DD2, and 7G8 culture strains were used as positive controls. The NEB high-fidelity PCR kit (New England BioLabs, USA) was used to amplify the genes according to the manufacturer’s instructions with a 50.0 μL master mix preparation using 5× buffer.

### Individual patient and pooled patient target amplicon deep sequencing.

The amplification products were sequenced using the Malaria Resistance Surveillance (MaRS) protocol (27). In brief, unique sequence indices were added to PCR amplicons for the individual samples and the pools using the Nextera XT kit (Illumina, USA). Two separate sequencing runs were performed, one for the individual samples and one for the pools.

### Statistical analysis.

Sequences were analyzed at 11 loci representing the major reportable SNPs for *pfdhfr* (A16V, N51I, C59R, S108N, and I164L) and *pfdhps* (I431V, S436A, S437G, K540E, A581G, and A613S). A sample was considered a sequencing success if at each locus the percent quality (Q) was 30 or higher and more than five reads were observed. SNPs were analyzed using a combination of Python 3.0 and Geneious with a multitier workflow approach, beginning with a quality control check for naming schema. Raw sequences were then imported into Geneious, where the workflow was as follows: Trimming, Map to reference, Find Variations/SNP. The annotated file containing all the information per sample was uploaded into Jupyter Notebook, where a report was created containing the variant allele frequency (VAF) for each individual sample or pool. The VAF at each polymorphic site was calculated using the following formula:

$$\text{Weighted VAF}=\overset{N}{\underset{i\;=\;1}{\sum }}\frac{\mathrm{VA}F_{i}w_{i}}{\sum_{i\;=\;1}^{N}w_{i}}$$

Here, $\mathrm{VA}F_{i}$ is the variant allele frequency for DNA sample *i*, $w_{i}$ is the weight for DNA sample *i*, and *N* is the total number of individual and pooled DNA samples for each province. For the individually sequenced samples, $w_{i}=1$, while for the pooled DNA samples, $w_{i}$ is the number of samples included in the pool. For each province, all day 0 samples and DoF samples from confirmed reinfections were included in this analysis, while the DoF samples from participants that were confirmed recrudescences were excluded from further analysis. Reinfections by definition are a new infection on the DoF, act as an independent observation of a parasite, and can be included in frequency estimates. In contrast, recrudescent infections on the DoF include the same strain present at baseline, and inclusion would be equivalent to double-counting these samples, biasing the frequency estimation. The results were not stratified by day 0 or DoF collection because SP was not expected *a priori* to be associated with clinical outcome in a trial of artemether-lumefantrine or artesunate-amodiaquine and because of the small overall number of treatment failures.

For individual samples, haplotypes were constructed if the major frequency allele occurred at more than 95% of reads for the *pfdhfr* and *pfdhps* loci of interest. Samples where this condition was not met were considered to have more than one strain present, precluding the inference of haplotypes.

### Ethics review.

The study was reviewed by human subject review boards at the Angolan Ministry of Health and the Centers for Disease Control and Prevention’s Center for Global Health (protocol 2014-233c). The parents or guardians of the study participants provided written informed consent.

### Data availability.

Data have been uploaded to the NCBI under SRA accession numbers SRR1530988-SRR15309198 and SRR19887632-SRR19887826 and BioProject accession number PRJNA428490.
