# Supplementary material for: Molecular Markers of Sulfadoxine-Pyrimethamine Resistance in Samples from Children with Uncomplicated Plasmodium falciparum at Three Sites in Angola in 2019
Source: Antimicrob Agents Chemother. 2023 Mar 14;67(4):e01601-22. doi: 10.1128/aac.01601-22 (PMC10112138; doi:10.1128/aac.01601-22)
Supplement: Supplemental file 1 — Supplemental material. Download aac.01601-22-s0001.docx, DOCX file, 0.2 MB [file aac.01601-22-s0001.docx]

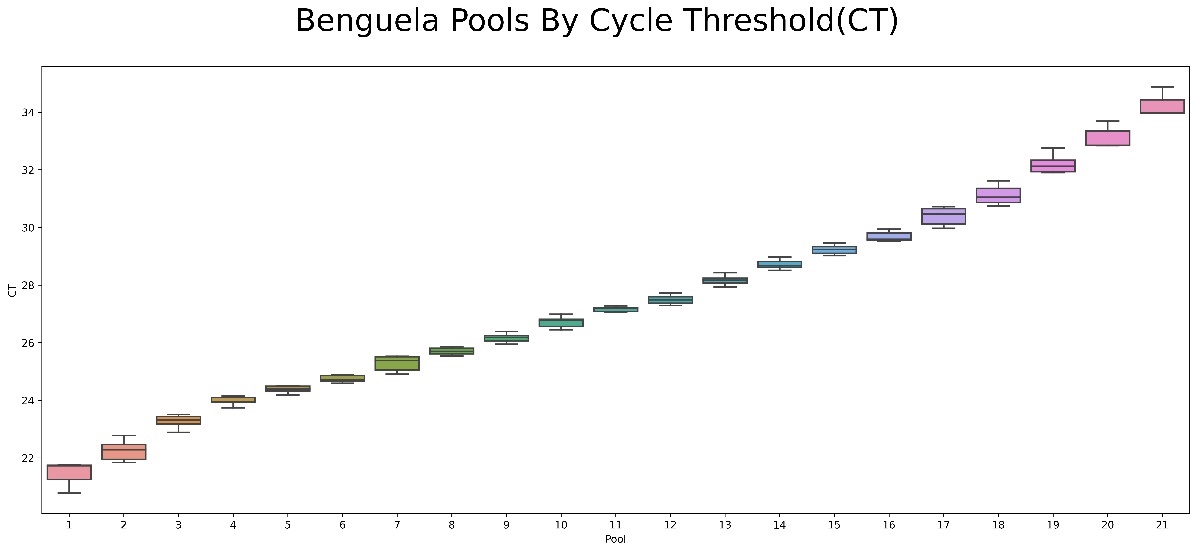


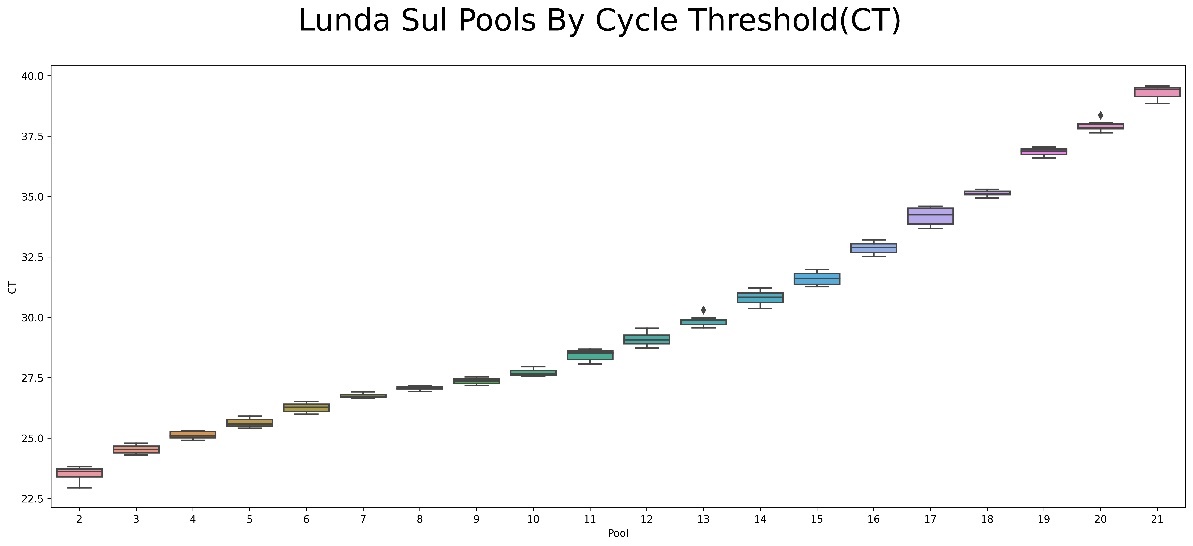


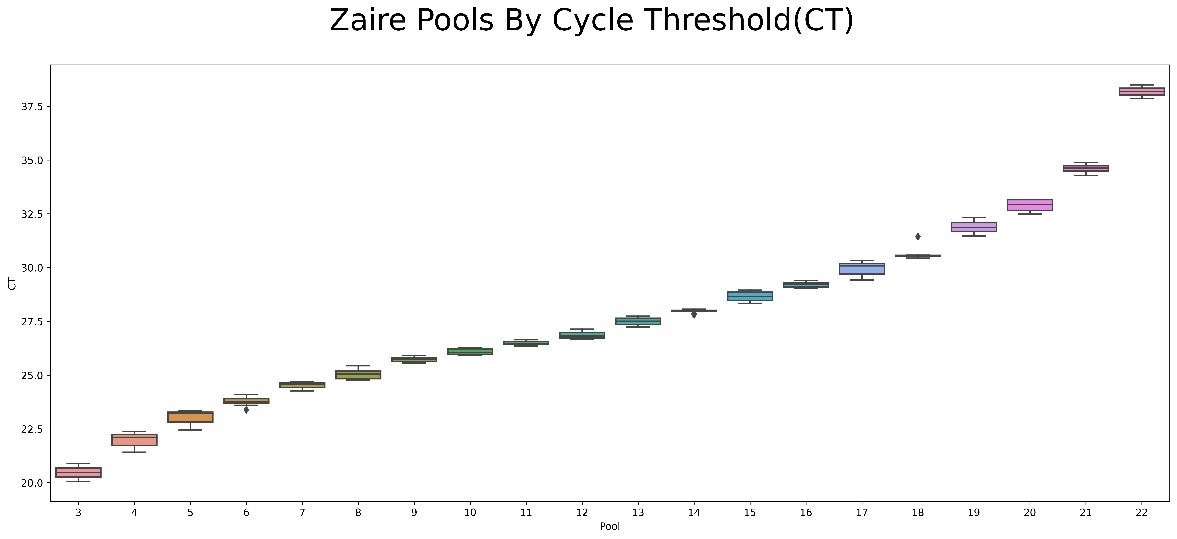


**Supplemental Figure S1**. Distribution of PET-PCR Ct values in pools designed from *P. falciparum* samples from Benguela, Lunda Sul, and Zaire Provinces, Angola 2019. Pools were constructed to include up to a maximum of 10 samples, and such that the difference in Ct values of any two samples in the pool did not exceed 1.
